# A new classification of the sagittal root positioning of the mandibular anterior teeth in relation to their anterior buccal bone using cone-beam computed tomography (CBCT)

**DOI:** 10.34172/japid.2020.014

**Published:** 2020-11-17

**Authors:** Omid Moghaddas, Irana Behravan

**Affiliations:** ^1^Department of Periodontology, Faculty of Dentistry, Tehran Islamic Azad Medical Sciences University, Tehran, Iran; ^2^Irana Behravan DMD, DDS, Tehran, Iran

**Keywords:** Classification, Cone-beam computed tomography, Tooth root diagnostic imaging

## Abstract

**Background:**

This study aimed to develop a classification for the sagittal root positioning (SRP) of mandibular anterior teeth in terms of their anterior buccal bone for use before placing immediate implants.

**Methods:**

A retrospective review of CBCT images was conducted on 150 patients (75 males and 75 females; mean age: 47.5 years) who met the inclusion criteria. The root position of the tooth samples was classified as buccal, middle, or lingual types according to their respective sagittal position and subtypes a, b, c, or d, according to the morphology of their osseous housing.

**Results:**

The frequencies of the root positions of each classified group of the sample teeth were as follows: 14% buccal type, 77% middle type, and 8% lingual type; 18.0% subtype a, 4.33% subtype b, 75.55% subtype c, and 2.11% subtype d. As a complementary procedure for data collection, the sagittal position of the apex was classified into Class I (buccally angulated apex: 4.6%), Class II (apex with no angulation: 78.2%), Class III (lingually angulated apex: 0.7%) and Class IV (exposed root: 16.3%). In addition, the results of the examination of the buccal undercut showed that in 1.6%, 32.0%, and 66.3% of the sample teeth, the undercut was located coronally, medially, and apically, respectively.

**Conclusion:**

Considering these results, the newly proposed SRP classification system can be used to study the mandibular anterior buccal bone morphology as a diagnostic tool for immediate implant treatment.

## Introduction

Due to the ever-increasing use of dental implants, the importance of a sagittal root position (SRP) for the most suitable treatment planning has increased in the anterior esthetic zone. Prognosis and the outcome of treatment are of great importance in immediate implants due to the odds of failure. Nevertheless, immediate implants are more prevalent since they can lead to tissue preservation and a shorter treatment period if they are placed correctly.^1-3^

A classification system for sagittal positioning of the root apex was suggested by Kan et al^4^ after studying the SRP of maxillary anterior teeth in their osseous housing.

Furthermore, a new classification system was introduced by Xu et al5 to determine changes in the thickness of the buccal bone of maxillary anterior teeth in terms of the SRP of maxillary central incisors.

The SRP of maxillary anterior teeth has been analyzed in previous studies; however, the SRP of mandibular anterior teeth has not been studied. To the best of our knowledge, this is the first report on the analysis of the SRP of mandibular anterior teeth relative to the buccal bone to introduce a new classification system for the anterior region of the maxilla and mandible.

## Methods

### Patients

This retrospective study was conducted on CBCT images of 900 mandibular anterior teeth in 150 patients with bilateral mandibular canines and lateral and central incisors. The patients consisted of 75 males and 75 females, with an age range of 10‒78 and a mean age of 47.5 years. All the patients were free of any form of oral infection, systemic disease, and history of orthodontic treatment and periodontal surgery.

### CBCT imaging

CBCT scans of patients were taken by a NewTom GiANO machine, using a single cross-sectional image (10 mm) in the panoramic view. The NNT software was used to analyze the CBCT scans.

The New Classification System for the Sagittal Root Position (SRP)

**Figure 1:**
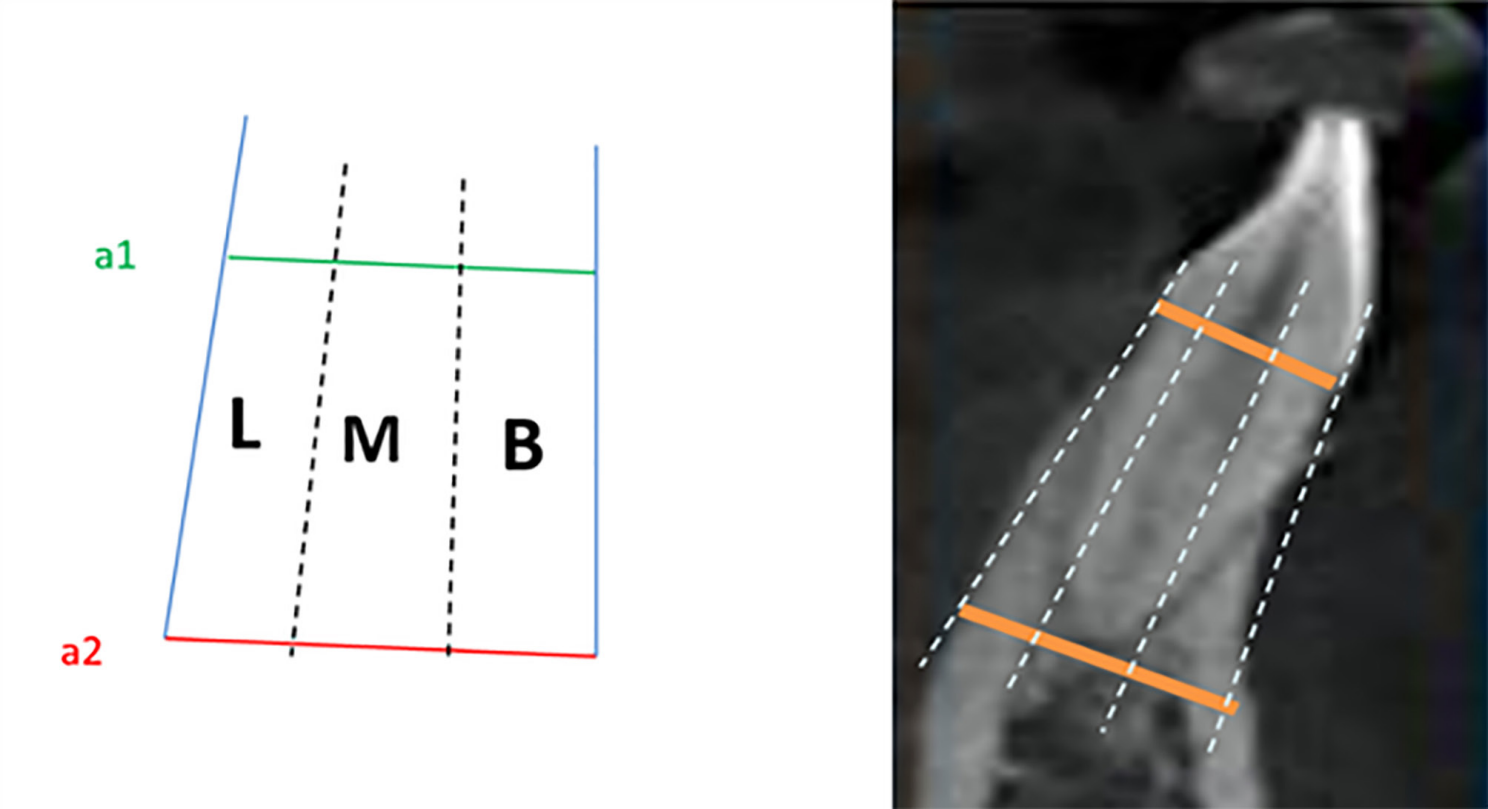
SRP classification – in this case the sample categorized in middle class

The SRP classification of Xu et al^5^ was adopted and modified to use for mandibular anterior teeth. On the sagittal CBCT of the six mandibular anterior teeth, lines a1 and a2 were drawn, so that line a1 passed through the highest points of the buccal and lingual alveolar bone between the outer margins of the buccal and lingual cortices; line a2 passed through the apical point of the tooth between the outer margins of the buccal and lingual cortices, parallel to line a1. Lines a1 and a2 were divided into three equal parts by four dots, which were connected by four lines from line a1 to line a2. The three regions formed between lines a1 and a2 were named buccal (B), medial (M), and lingual (L) (Figure 1). The SRP of the tooth samples in the alveolar bone was classiﬁed as buccal (B), medial (M), and lingual (L) type according to the position of the root in the three drawn sections.

**Figure 2:**
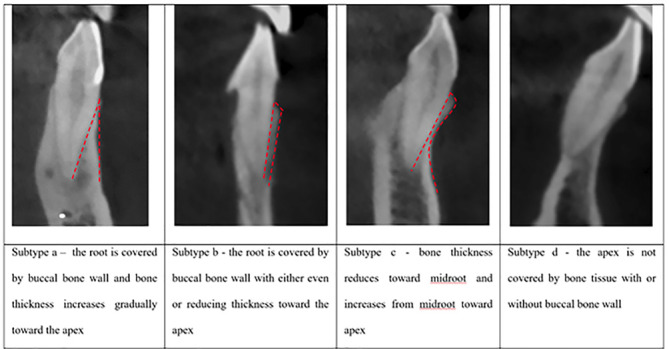
SRP subtypes a/b/c/d.

Each SRP type was classified into four subtypes, referred to as a, b, c, and d according to the morphology of the buccal bone (Figure 2).

**Figure 3:**
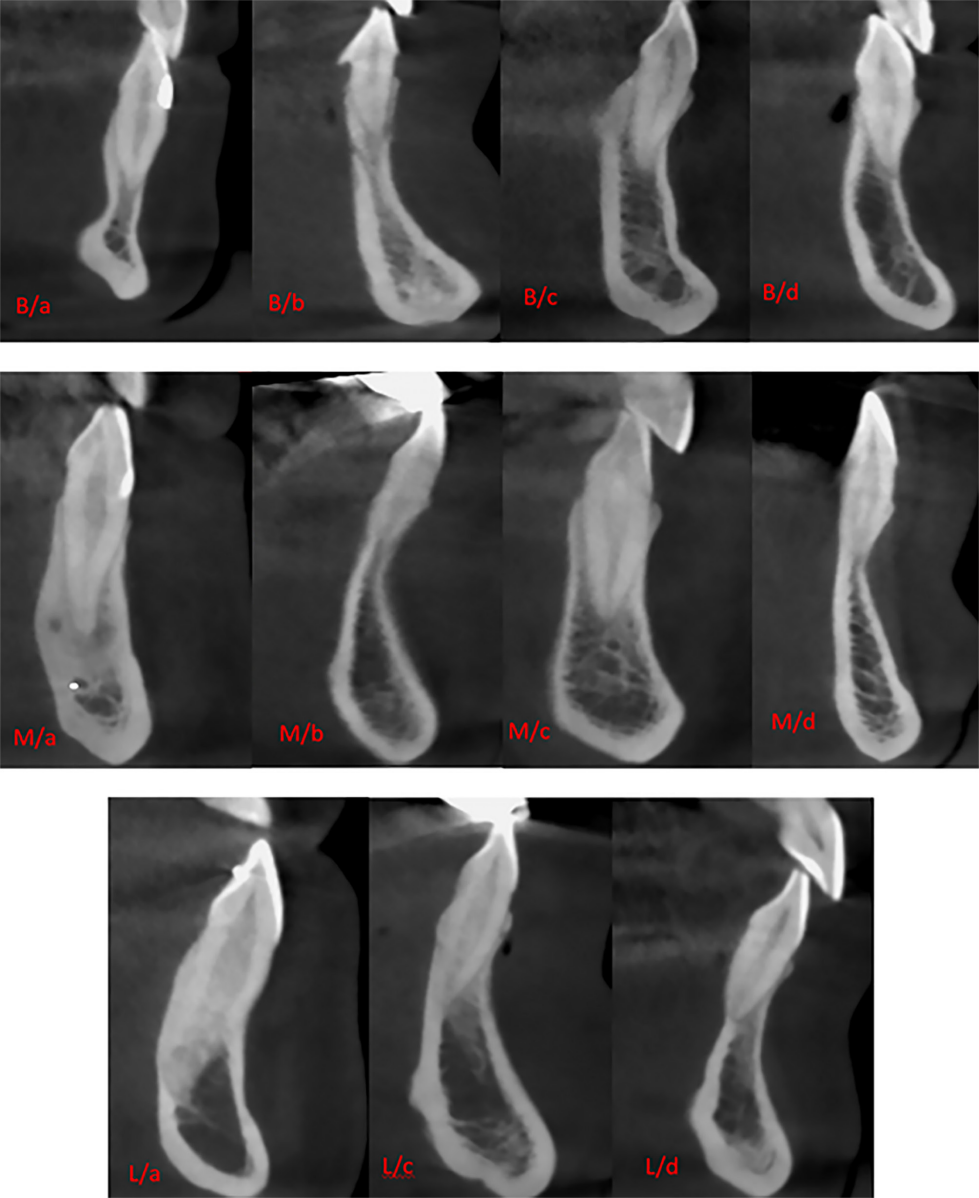
SRP classification - B/a- Class B subtype a, B/b- Class B subtype b, B/c- Class B subtype c, B/d- Class B subtype d, M/a- Class M subtype a, M/b- Class M subtype b, M/c- Class M subtype c, M/d- Class M subtype d, L/a- Class L subtype a, L/c- Class L subtype c, L/d- Class L subtype d.

In subtype a, the root is covered by a buccal bone wall, and bone thickness increases gradually toward the apex. In subtype b, the root is covered by a buccal bone wall, and the thickness of the bone decreases or remains unchanged toward the apex. In subtype c, bone thickness decreases toward the mid-root and increases from the mid-root toward the apex. In subtype d, the apex is not covered by bone, with or without a buccal bone wall (Figures 2 and 3). The proposed subtypes are different from the classification proposed by Xu et al^5^ due to the morphological differences of buccal bone in the maxilla and mandible. In some cases, the mandibular buccal bone has a more pronounced thickness change by decreasing toward the mid-root and increasing toward the apex, which is absent in the maxilla; therefore, it was not included in the classification of Xu et al.

### Reliability assessment

The buccal bone thickness was measured in three levels (coronal, middle, and apical thirds) of the root covered by the alveolar bone to ensure the bone thickness changes along the root.

### Classification of the Sagittal Positioning of the Apex^4^

**Figure 4:**
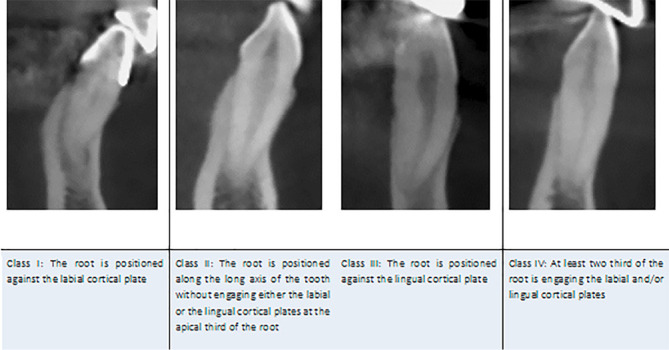
Kan’s classification of apex sagittal positioning.

The apex position was classified as classes I, II, III, and IV according to the sagittal inclination of the apex of the tooth long axis (Figure 4). In the class I category, the root is positioned against the labial cortical plate. In the class II category, the root is positioned along the long axis of the tooth without engaging either the labial or lingual cortical plate at the apical third of the root. In the class III category, the root is positioned against the lingual cortical plate, and if at least two-thirds of the root engage the labial and/or lingual cortical plate, it is classified as class IV (Figure 4).

### The buccal undercut depth and location

**Figure 5:**
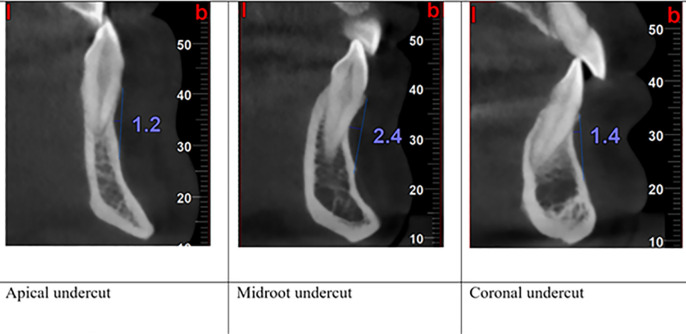
Undercut sagittal location.

To measure the undercut depth, the distance of the deepest point (most posterior) of the buccal plate from a line connecting the most anterior points of the buccal bone was measured for each tooth, and according to its location, the undercut was classified as coronal, middle, or apical (Figure 5).

### Statistical analysis

The data were presented as percentages or frequencies and compared using one-way ANOVA, followed by post hoc tests. Statistical analyses were performed using SPSS. Statistical significance was set at P<0.05.

## Results

In this study, 900 mandibular anterior teeth (300 central incisors, 300 lateral incisors, and 300 canines) were examined in 150 patients (50% males and 50% females) with a mean age of 47±13.48 and an age range of 19–70 years.

Table 1 shows the mean buccal bone thicknesses (coronal, middle, and apical thirds of the root), root lengths in bone, and undercut depths.

**Table 1:** Descriptive variables according to tooth type

**Tooth type**	**Central**	**Lateral**	**Canine**
**Average height of root in bone**	9.46 ± 1.73	10.9 ± 1.77	13.05 ± 2.14
**Average coronal buccal bone thickness**	1.0 ± 0.46	0.97 ± 0.45	0.83 ± 0.56
**Average midroot buccal bone thickness**	0.84 ± 0.64	0.63 ± 0.58	0.53 ± 0.58
**Average apical buccal bone thickness**	2.06 ± 0.99	2.03 ± 0/99	2.49 ± 1.32
**Average undercut depth**	1.88 ± 0.52	1.92 ± 0.54	1.62 ± 0.57

The highest mean length of the root in the alveolar bone was 13.5 mm in the canines, and the lowest mean was 9.46 mm in the central incisors. The difference in the root length of the three teeth was statistically significant (P<0.05); however, it was not clinically significant.

The highest mean buccal bone width in the coronal region was in central incisors with 1.00 mm, with the lowest in canines with 0.83 mm. The test showed that the coronal width difference in the teeth was statistically significant (P<0.01). However, there was no significant difference between the central and lateral incisors (P<0.7).

The highest mean buccal bone width in the middle third was in the central incisors with 0.84 mm, and the lowest was in the canines with 0.53 mm, which was statistically significant (P<0.01).

The highest mean buccal bone width in the apical third was in the canines with 2.49 mm, with the lowest in the central and lateral incisors with 2.06 and 2.3 mm, respectively. This difference was statistically significant (P<0.01); however, there was no significant difference between the central and lateral incisors.

The highest mean depth of the buccal undercuts was in the lateral incisors with 1.92 and 1.88 mm in the central incisors, and the lowest mean depth was in the canines with 1.62 mm. The difference was statistically significant (P<0.01).

The distribution of teeth in terms of the new proposed classification of the sagittal root position (SRP) and its subtypes by gender and the tooth type are presented in Table 2.

**Table 2:** Frequency distributions of SRP classification in terms of gender and tooth type

**Gender**	**Male**				**Female**	
**Tooth type**	**Central**	**Lateral**	**Canine**	**Central**	**Lateral**	**Canine**
B/a	2 (16.7%)	3 (21.4%)	4 (18.2%)	1 (6.3%)	0	11 (24.4%)
B/b	4 (33.3%)	2 (14.3%)	4 (18.2%)	5 (31.3%)	7 (41.2%)	4 (8.9%)
B/c	6 (50.0%)	8 (57.1%)	12 (54.5%)	10 (62.5%)	10 (58.8%)	23 (51.1%)
B/d	0	1 (7.1%)	2 (9.1%)	0	0	7 (15.6%)
M/a	15 (12.4%)	11 (8.9%)	43 (34.7%)	13 (10.9%)	11 (9.3%)	29 (28.4%)
M/b	5 (4.1%)	2 (1.6%)	1 (0.8%)	0	2 (1.7%)	3 (3.9%)
M/c	100 (82.6%)	111 (89.5%)	80 (64.5%)	105 (88.2%)	104 (88.1%)	70 (86.6%)
M/d	1 (0.8%)	0	0	1 (0.8%)	1 (0.8%)	0
L/a	3 (17.6%)	3 (25.0%)	2 (50.0%)	5 (33.3%)	4 (26.7%)	2 (66.7%)
L/b	0	0	0	0	0	0
L/c	13 (76.5%)	9 (75.0%)	2 (50.0%)	7 (46.7%)	9 (60.0%)	1 (33.3%)
L/d	1 (5.9%)	0	0	3 (20.0%)	2 (13.3%)	0

In the SRP classification, 14.0% of the roots were positioned buccally (B/a, 17.4%; B/b, 20.6%; B/c, 54.9%; and B/d, 7.9%), with 77.0% medially (M/a, 17.5%; M/b, 1.8%; M/c, 81.7%; and M/d, 0.4) and 8.0% lingually (L/a, 24.6%; L/b, 0%; L/c, 53.2%; and L/d, 7/7%).

The most frequent subtype was subtype c, with 75%. The samples of the subgroups b and d were very small and comprised approximately 5% of the total samples. In this study, no sample was found with a lingual subtype b.

In the SRP classification, the a, b, c, and d subtypes in all the B, M, and L classes were similar in terms of number and percentage in both males and females and tooth types (P<0.04).

Table 3 presents the distribution of teeth by Kan’s classification in terms of the patients’ gender and the tooth type.

**Table 3:** Frequency distributions of apex sagittal position in terms of gender and tooth type based on Kan’s classification

**Gender**	**Men**			**Women**		
**Tooth type**	**Central**	**Lateral**	**Canine**	**Central**	**Lateral**	**Canine**
**I**	4 (2.7%)	4 (2.7%)	17 (11.3%)	2 (1.3%)	3 (2.0%)	12 (0.8%)
**II**	120 (80.0%)	122 (81.3%)	108 (72.0%)	123 (82.0%)	128 (85.3%)	103 (103%)
**III**	0	0	1 (0.7%)	0	1 (0.7%)	5 (3.3%)
**IV**	26 (17.3%)	24 (16.0%)	24 (16.0%)	25 (16.7%)	18 (12.0%)	30 (20.0%)

The apex was positioned buccally (Class I) in 4.6% of the samples, along the long axis of the tooth (Class II) in 78.2%, lingual (Class III) in 0.7%, and Class IV in 16.3% of the cases.

There were no significant differences in the patients’ gender and tooth types (P<0.04).

The distribution of samples based on the location of the undercuts in terms of the patients’ gender and tooth types is presented in Table 4.

**Table 4:** Frequency distributions of undercut location in terms of gender and tooth type

**Gender**	**Men**			**Women**		
**Tooth type**	**Central**	**Lateral**	**Canine**	**Central**	**Lateral**	**Canine**
**Coronal third**	117 (78.0%)	112 (74.7%)	108 (72.0%)	100 (66.7%)	78 (52.0%)	82 (54.7%)
**Mid-root**	29 (19.3%)	36 (24.0%)	42 (28.0%)	48 (32.0%)	70 (46.7%)	63 (42.0%)
**Apical third**	4 (2.7%)	2 (1.3%)	0	2 (1.3%)	2 (1.3%)	5 (3.3%)

The undercut was located coronally in 1.6% of the samples (the lowest frequency), medially in 32.0% of the cases, and apically in 66.3% (the highest frequency).

The results did not differ signiﬁcantly between the male and female subjects (P<0.2).

## Discussion

The alveolar buccal bone in the mandible’s anterior region is relatively thin and undergoes rapid resorption during the healing procedure compared to the lingual plate. The results of this study on the anterior mandibular teeth showed that buccal bone thickness decreased in 81.7% of the cases from the CEJ to the mid-root and increased toward the apex, unlike the maxilla in which it mostly increases toward the apex. This difference in thickness change was also noted previously by Kim et al.^6^ However, it is clear that medially located roots in the buccolingual plane have a better prognosis in immediate implant placement, regardless of bone thickness, due to the presence of both the buccal and lingual bone walls. The implant must be placed as medially as possible in buccally and lingually located root areas.^4,5^

According to Witek et al,^7^ the rotation of mandibular incisors and canines does not affect the thickness of the surrounding bone. However, it is necessary to evaluate the thickness of the bone, especially in the apical and labial cortical areas that are more prone to bone resorption after tooth extraction, and to make sure that the apex position is also specified.^8^ Compared to the mandibular and maxillary anterior teeth, it was shown that approximately 60.1% of the roots in the mandible had apices located along the long axis of the tooth. However, in the maxilla, the majority of roots had buccally inclined apices.^4,9^

The undercuts can also affect bone thickness and increase the risk of bone fenestration, collapse, and faster resorption, especially around the inclined apices.^9-11^ Since more than two-thirds of mandibular anterior teeth had undercut depths >1 mm, with none exhibiting <0.5-mm-deep undercuts, obligatory treatments, such as ridge augmentation, bone grafts, etc., must be rendered during implant placement to restore and augment bone thickness. In this context, flapless placement of implants in the mandibular anterior region might be at a higher risk of failure if managed without accurate evaluation and guided surgery.^12,13^ Determining the location of undercuts in this study showed that 66.3% of the undercuts were located apically, enabling the dentists and patients to select alternative treatments, such as bone augmentation.^2,14^

## Conclusion

This research proposes a new SRP classification system to study mandibular anterior buccal bone morphology as a diagnostic tool for immediate implant treatment. Since more than two-thirds of mandibular anterior teeth had undercut depths over 1 mm and none with less than 0.5-mm-deep undercuts, obligatory treatments, such ridge augmentation, bone graft, etc., must be rendered during implant placement to restore and augment bone thickness, indicating that flapless placement of implants in the mandibular anterior region might be at a higher risk of failure if managed without accurate evaluation and guided surgery.

## Abbreviations

SRP (sagittal root position): The position of the root of the anterior teeth in the alveolar housing in the sagittal plane.

CBCT (cone-beam computed tomography) image: A medical imaging technique consisting of divergent x-ray computed tomography, forming a cone.

## Authors’ contributions

MO: Conceptualization, IB: Formal Analysis, MO, IB: Investigation, MOand IB: Methodology, IB, MO: Project Administration, IB: Writing–Original Draft, MO, IB: Writing–Review&Editing. All authors have read and approved the final manuscript.

## Competing interests

The authors declare that there were no competing interests between the authors.

## Ethics Approval

The study was conducted in an in-vitro environment and no humans participated in the study. The study did not have any ethical registrations.
